# Characterizing the Link between Glycosylation State and Enzymatic Activity of the Endo-β1,4-glucanase KORRIGAN1 from Arabidopsis thaliana*

**DOI:** 10.1074/jbc.M113.475558

**Published:** 2013-06-19

**Authors:** Eva Liebminger, Josephine Grass, Friedrich Altmann, Lukas Mach, Richard Strasser

**Affiliations:** From the Departments of ‡ Applied Genetics and Cell Biology and; § Chemistry, BOKU, University of Natural Resources and Life Sciences, Muthgasse 18, A-1190 Vienna, Austria

**Keywords:** Arabidopsis, Glycobiology, Glycosylation, Plant Biochemistry, Plant Cell Wall, Post Translational Modification, Cellulose Synthesis

## Abstract

Defects in *N*-glycosylation and *N*-glycan processing frequently cause alterations in plant cell wall architecture, including changes in the structure of cellulose, which is the most abundant plant polysaccharide. KORRIGAN1 (KOR1) is a glycoprotein enzyme with an essential function during cellulose biosynthesis in *Arabidopsis thaliana*. KOR1 is a membrane-anchored endo-β1,4-glucanase and contains eight potential *N*-glycosylation sites in its extracellular domain. Here, we expressed *A. thaliana* KOR1 as a soluble, enzymatically active protein in insect cells and analyzed its *N*-glycosylation state. Structural analysis revealed that all eight potential *N*-glycosylation sites are utilized. Individual elimination of evolutionarily conserved *N*-glycosylation sites did not abolish proper KOR1 folding, but mutations of Asn-216, Asn-324, Asn-345, and Asn-567 resulted in considerably lower enzymatic activity. In contrast, production of wild-type KOR1 in the presence of the class I α-mannosidase inhibitor kifunensine, which abolished the conversion of KOR1 *N*-glycans into complex structures, did not affect the activity of the enzyme. To address *N*-glycosylation site occupancy and *N*-glycan composition of KOR1 under more natural conditions, we expressed a chimeric KOR1-Fc-GFP fusion protein in leaves of *Nicotiana benthamiana*. Although Asn-108 and Asn-133 carried oligomannosidic *N*-linked oligosaccharides, the six other glycosylation sites were modified with complex *N*-glycans. Interestingly, the partially functional KOR1 G429R mutant encoded by the *A. thaliana rsw2-1* allele displayed only oligomannosidic structures when expressed in *N. benthamiana*, indicating its retention in the endoplasmic reticulum. In summary, our data indicate that utilization of several *N*-glycosylation sites is important for KOR1 activity, whereas the structure of the attached *N*-glycans is not critical.

## Teaser

**Background:** *N*-Glycosylation affects the biosynthesis of the endo-β1,4-glucanase KORRIGAN1 which is crucial for cell wall synthesis in plants.

**Results:** Underglycosylated KORRIGAN1 displays reduced enzymatic activity. This is not observed upon changes to the *N*-glycan composition.

**Conclusion:** Modified *N*-glycan processing exerts no direct effect on KORRIGAN1 function *in planta*.

**Significance:** The relevance of several *N*-glycosylation sites for KORRIGAN1 activity is revealed.

## Introduction

Glycosylation of proteins is a central co- and post-translational modification of secretory and membrane-bound proteins in all eukaryotes. *N*-Glycans covalently attached to asparagine residues have a vast number of diverse functions including support of protein folding, quality control processes, protein targeting, and modulation of protein-protein interactions (1). In plants, several *N*-glycan biosynthesis and processing mutants have been identified with defects in synthesis of cellulose (2–5), which is a major component of the plant cell wall. Consequently, it has been proposed that one or several proteins involved in cellulose biosynthesis are glycosylated, and abolished *N*-glycosylation or changes of their *N*-glycan structures could result in altered cellulose content or composition (6). The *Arabidopsi thaliana* cellulose synthase proteins that form the functional multisubunit cellulose synthase complex contain several potential *N*-glycosylation sites. However, due to the proposed membrane protein topology, most of these *N*-glycosylation sites do not face the lumenal or extracellular space (7) and are therefore not accessible for the oligosaccharyltransferase that initiates protein glycosylation by transfer of the preassembled oligosaccharide precursor in the endoplasmic reticulum (ER).^2^ This hypothesis is also supported by experimental data (3). Among other *A. thaliana* proteins implicated in cellulose biosynthesis, the membrane-bound endo-β1,4-glucanase KORRIGAN1 (KOR1) is a possible candidate glycoprotein that could be directly affected by changes in *N*-glycosylation. The precise molecular function of KOR1 and its orthologs from other species during synthesis of cellulose is still unclear (8–10). *A. thaliana* mutants partially deficient in KOR1 exhibit reduced cellulose content in roots and minor changes in other cell wall polysaccharides as well as cell elongation defects (11–16). Close-to-null mutations of KOR1 display also defects in cytokinesis and severe morphological abnormalities (17). KOR1 is a type II membrane protein with eight predicted *N*-glycosylation sites in its extracellular domain (11), and most of these *N*-glycosylation sites are conserved in different KOR1 orthologs from other species (18, 19). The catalytic domain of *Brassica napus* Cel16, which shares 94% sequence identity with *A. thaliana* KOR1 was found to be heavily glycosylated when expressed in *Pichia pastoris* (20). Moreover, deglycosylation with endoglycosidases almost completely abolished the endoglucanase activity of Cel16, indicating that *N*-glycosylation might play a crucial role for KOR1 enzymatic activity. Similarly, the enzymatic activity of recombinant PttCel9A, a KOR1 homolog from *Populus tremula x tremuloides*, was reduced by 80% when subjected to deglycosylation (18). Altered mobility upon SDS-PAGE separation and reduced accumulation of endogenous KOR1 in different *A. thaliana* glycosylation mutants further indicated that changes in *N*-glycosylation might directly affect KOR1 function, leading to the observed defects in cellulose biosynthesis and cell elongation (21, 22). These findings are also corroborated by genetic evidence showing drastically enhanced root growth phenotypes of the weak *kor* allele *rsw2-1* in *cgl1 rsw2-1*, *stt3a rsw2-1*, *mns3 rsw2-1*, and *ost3/6-1 rsw2-1* double mutants, which all display alterations in *N*-glycosylation (21–23). In particular, the enhanced phenotype of *cgl1 rsw2-1* is of interest as the formation of complex *N*-glycans is completely blocked in this mutant. Consequently, it has been proposed that KOR1 requires complex *N*-glycans for its *in vivo* function in plants (21, 24). Although these studies support the idea that *N*-glycosylation of KOR1 and processing of *N*-glycans on KOR1 are crucial for its biological function, the precise role of individual *N*-glycans on KOR1 and their contribution to the enzymatic activity of KOR1 are still unclear.

To characterize the relevance of *N*-glycans for KOR1 function in more detail, we expressed the catalytic domain of wild-type *A. thaliana* KOR1 and various mutants lacking selected *N*-glycosylation sites in insect cells. We assessed the glycosylation status of the recombinant KOR1 variants by MS and report that KOR1 is glycosylated on all eight potential *N*-glycosylation sites. Moreover, the effect of *N*-glycan elimination on the enzymatic activity of recombinant KOR1 and the role of individual *N*-glycans for KOR1 synthesis in plants was examined. Our data firmly establish that proper *N*-glycosylation of KOR1 is indeed important for its enzymatic activity but suggest that *N*-glycan processing to complex *N*-glycans is dispensable for KOR1 function in plants.

## EXPERIMENTAL PROCEDURES

### 

#### 

##### Expression of KOR1 in Insect Cells

The KOR1 open reading frame lacking the N-terminal cytoplasmic region and transmembrane domain was amplified from *A. thaliana* leaf cDNA with Phusion® High-fidelity DNA polymerase (New England Biolabs) using the primers KOR1–6F (TATAGCGGCCGCCTTGATCGTTAAAACTGTGCCGC) and KOR1–7R (TATAGAATTCAAGGTTTCCATGGTGCTGGTG) and cloned into NotI- and EcoRI-digested baculovirus transfer vector pVTBacHis-1 (25). In the resulting construct, the amino acid sequence is placed downstream of the melittin signal peptide, a His_6_ tag, and an enterokinase cleavage site. Cultivation of *Spodoptera frugiperda* Sf21 cells, baculovirus-mediated infection in the absence or presence of 2 μm kifunensine (Sigma) and purification of the recombinant proteins were performed as described previously (26). At the end of the purification procedure, the KOR1-containing fractions were pooled and concentrated by ultrafiltration to ∼ 1 mg/ml protein after a buffer exchange to 50 mm MES (pH 6.0), 250 mm NaCl, 30 mm CaCl_2_ by means of diafiltration.

##### Site-directed Mutagenesis of rKOR1

Mutations in several *N*-glycosylation sites of KOR1 were introduced using the QuikChange site-directed mutagenesis kit (Stratagene) and Phusion® high-fidelity DNA polymerase. Mutagenesis was performed following the instructions of the manufacturer. The pVTBacHis-KOR1 construct was used as templates for site-directed mutagenesis, and the respective single *N*-glycosylation mutants were generated using the following primers: N216Q-F, CTTTCTCAAGACTTTCCAAAGTACTGCTGATTCC and N216Q-R, GGAATCAGCAGTACTTTGGAAAGTCTTGAGAAAG; N324Q-F, TCTAGCAAGTTCTATCAGTCAAGTATGTATTGG and N324Q-R, CCAATACATACTTGACTGATAGAACTTGCTAGA; N345Q-F, TATTATGCTACCGGACAAGTAACGTATCTCAAT and N345Q-R, ATTGAGATACGTTACTTGTCCGGTAGCATAATA; N408Q-F, GACTATGCTGGTCTGTTGGTGGAAGGTCCTTAG and N408Q-R, CTAAGGACCTTCCACCAACAGACCAGCATAGTC; N425Q-F, GCCTATTTTCAACAAATTTCAAAGAACCAATGGAGGTTTAA and N425Q-R, TTAAACCTCCATTGGTTCTTTGAAATTTGTTGAAAATAGGC; and N567Q-F, TGTCCGTATGAACTACCAATACACTGAACCGACTC and N567Q-R, GAGTCGGTTCAGTGTATTGGTAGTTCATACGGACA. The single mutant N324Q was used as template to generate the double mutant N324Q/N345Q, and this double mutant was subsequently used as template to obtain the triple mutant N216Q/N324Q/N345Q. To confirm the introduced mutations, all constructs were subjected to DNA sequencing using the following primers: for N216Q and N216Q/N324Q/N345Q primer, KOR1–3F (TTTGGTGGGAGGTTATTATGATGC); for N324Q, N345Q, and N324Q/N345Q primer, KOR1–14F (TGGACTATAAAAGGCCCGTGACTA); and for N408Q, N425Q, and N567Q, primer KOR1–12F (CAAGAAACCAAACCCAAACACG).

##### Plant Materials

*A. thaliana* ecotype Col-0 was used as a wild-type control. The *rsw2-1* seeds (14) were obtained from the European *Arabidopsis* Stock Centre, and the *fut11 fut12* double knock-out line was described previously (27). All plants were grown under long day conditions at 22 °C. *Nicotiana benthamiana* plants were grown under long day conditions at 24 °C. Protein extraction and immunoblotting was carried out as described previously (23).

##### Transient Expression of KOR1 in N. benthamiana

The KOR1 coding region was amplified using the following primers: KOR1–4F, TATATCTAGAATGTACGGAAGAGATCCATGGGG and KOR1–5R, TATAGGATCCAGGTTTCCATGGTGCTGGTGGAG; and the PCR fragment was subcloned using a Zero Blunt® TOPO PCR cloning kit (Invitrogen). Subsequently, KOR1 was excised and ligated into the XbaI/BamHI digested binary expression vector p29-Fc-GFP. p29 is derived from p27 (28) and contains an enhanced CaMV35S promoter derived from vector pVKH18En6 (29) and the Fc-GFP domains for purification and subcellular localization (30). Mutated versions of KOR1 were generated using site-directed mutagenesis as described above. Transient expression in *N. benthamiana* wild-type and ΔXTFT lines (31) was done by infiltration of leaves with agrobacteria containing the binary expression vectors as described previously (30). Infiltrations were carried out with agrobacteria diluted to an *A*_600_ of 0.2, and infiltrated leaf material was harvested 48–72 h post infiltration. KOR1 was purified from 800 mg of infiltrated leaves by incubation with rProtein A-Sepharose (GE Healthcare) as described previously (30). To generate KOR1_RSW2–1_-Fc-GFP, the respective KOR1 DNA fragment was amplified as described above from cDNA derived from *A. thaliana rsw2-1* plants. The presence of the mutation that leads to the G429R change (14, 15) was confirmed by DNA sequencing.

##### LC-ESI-MS Analysis of Tryptic Glycopeptides

To analyze the *N*-glycans of purified recombinant KOR1, proteins were separated by SDS-PAGE (10%) under reducing conditions and detected by Coomassie Brilliant Blue staining. The corresponding band was excised from the gel, destained, carbamidomethylated, in-gel digested with trypsin/chymotrypsin, and analyzed by LC-ESI-MS as described recently (32, 33).

##### Enzymatic Deglycosylation and Immunoblot Analysis

Purified KOR1 or total protein extracts were subjected to enzymatic deglycosylation as described in detail recently (34). Deglycosylated proteins were then either analyzed by LC-ESI-MS or were subjected to SDS-PAGE (10%) followed by immunoblot analysis with anti-GFP-HRP (Miltenyi Biotec), anti-human IgG (Promega), or anti-KOR1 antibody (raised in rabbits against the synthetic peptide CSGEEEATGKIDKNT; Genscript). The detection was performed with Super Signal West Pico chemiluminescent substrate (Pierce).

##### KOR1 Activity Assays

Solutions (10 μl) of purified recombinant KOR1 (rKOR1) produced in insect cells (0.35–4 μg) in 50 mm MES (pH 6.0), 250 mm NaCl, and 30 mm CaCl_2_ were incubated for 60 min at 30 °C with 90 μl of 0.1% (carboxymethyl)cellulose 4M (Megazyme) resuspended in the same buffer. The reaction was stopped by the addition of 400 μl of ice-cold 50 mm sodium borate (pH 10.0). 500 μl of freshly prepared 2,2′-bicinchoninic acid solution (a mixture of equal volumes of reagent A (5 mm 2,2′-bicinchoninic acid, 512 mm Na_2_CO_3_, 288 mm NaHCO_3_) and reagent B (5 mm CuSO_4_, 12 mm l-serine)) was added, and the samples were incubated for 15 min at 95 °C. Samples were cooled on ice and then centrifuged for 3 min prior to measurement of their absorbance at 562 nm. Samples containing heat-inactivated enzyme were used as controls. The amount of reducing ends generated was then deduced using a glucose standard curve (0–100 nmol). All assays were done in duplicates. Enzymatic deglycosylation of KOR1 under non-reducing conditions prior to activity assays was performed without (control) or with endoglycosidase H (Endo H) (50,000 units/ml) or peptide *N*-glycosidase F (PNGase F) (50,000 units/ml) in 50 mm MES (pH 6.0), 250 mm NaCl, 30 mm CaCl_2_ for 60 min at 30 °C. Activity assays were then performed as outlined above. To analyze enzyme stability, rKOR1 proteins were incubated for 30 to 240 min at 30 °C prior to activity tests. Statistical analyses were performed using Student's *t* test, with *p* < 0.05 considered significant.

## RESULTS

### 

#### 

##### KOR1 Expressed in Insect Cells Is Heavily Glycosylated

**FIGURE 1.**
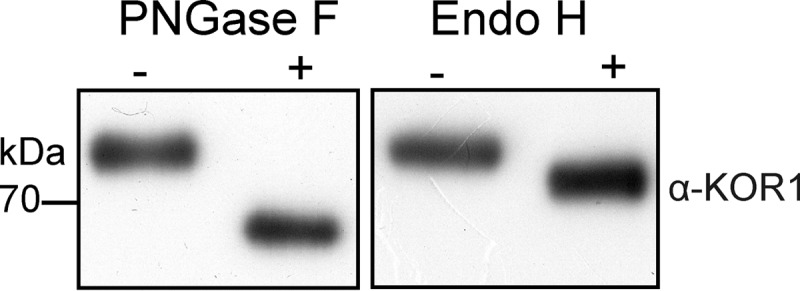
**Recombinant KOR1 is *N*-glycosylated.** rKOR1 WT was expressed in insect cells, purified from culture supernatants, and subjected to deglycosylation with PNGase F and Endo H under denaturing conditions. Proteins were separated by SDS-PAGE followed by immunoblotting with anti-KOR1 antibodies.

**FIGURE 2.**
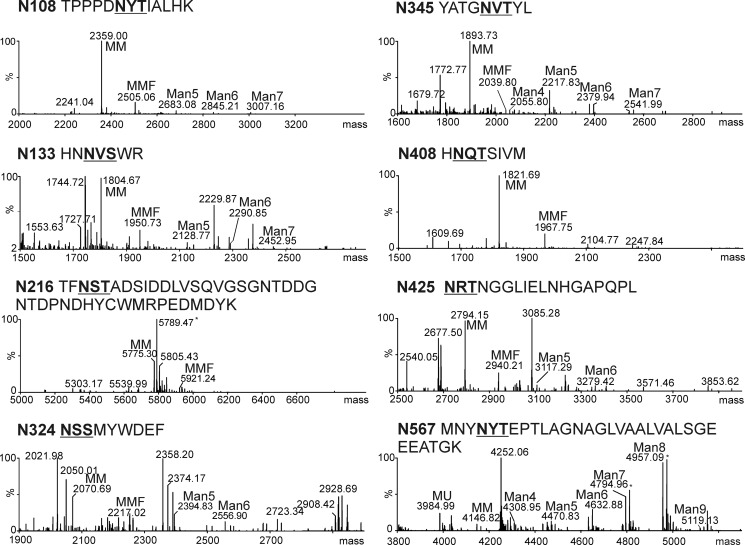
**All eight *N*-glycosylation sites of recombinant rKOR1 harbor glycans.** Spectra were obtained by LC-ESI-MS analysis of trypsin/chymotrypsin double-digested rKOR1 WT purified from insect culture supernatants. The major peaks with masses corresponding to individual *N*-glycan structures attached to the specified peptide are labeled. *N*-glycosylation sites are shown in *bold* and *underlined*. Sodium adducts are indicated with an *asterisk. MU*, Man_2_GlcNAc_2_; *MM*, Man_3_GlcNAc_2_; *MMF*, Man_3_FucGlcNAc_2_; *Man4* to *Man9*, Man_4_GlcNAc_2_ to Man_9_GlcNAc_2_. *N108*, Asn-108; *N133,* Asn-133; *N216,* Asn-216; *N324,* Asn-324; *N345,* Asn-345; *N408,* Asn-408; *N425,* Asn-425; *N567,* Asn-567.

The extracellular domain of *A. thaliana* KOR1 contains eight potential *N*-glycosylation sites (Asn-*X*-Ser/Thr, *X* can be any amino acid except proline). To analyze the role of *N*-glycosylation for KOR1 enzymatic activity we expressed an *A. thaliana* KOR1 variant lacking the *N*-terminal 90 amino acids (which include the cytoplasmic tail and the single transmembrane domain) in *S. frugiperda* Sf21 cells using a baculovirus expression system. Immunoblot analysis of cell extracts and culture supernatants revealed that recombinant wild-type KOR1 (rKOR1 WT) was successfully expressed in Sf21 cells and secreted into the culture medium. The secreted His_6_-tagged rKOR1 protein was purified by means of nickel-chelate affinity chromatography and subjected to enzymatic deglycosylation with Endo H, which cleaves oligomannosidic *N*-glycans, and PNGase F, which cleaves oligomannosidic and complex *N*-glycans lacking core α1,3-fucose (35), followed by immunoblotting with anti-KOR1 antibody. Both deglycosylation reactions led to distinct mobility shifts of rKOR1 WT, indicating that it is decorated with oligomannosidic and processed complex or paucimannosidic *N*-glycans (Fig. 1). To gain a more detailed insight into the glycan structures present on each *N*-glycosylation site of rKOR1 WT, glycopeptides derived from proteolytic digestion were analyzed by LC-ESI-MS. Mass spectrometry revealed that all eight *N*-glycosylation sites are occupied with *N*-glycans. Except for the peptide with glycosylation site Asn-567, which harbored mainly oligomannosidic *N*-glycans (in particular Man_8_GlcNAc_2_), the predominant structures present on the other seven *N*-glycosylation sites were Golgi-processed paucimannosidic *N*-glycans (Man_3_GlcNAc_2_ and Man_3_FucGlcNAc_2_). Only minor peaks with masses that match to oligomannosidic *N*-glycans were found (Fig. 2).

##### KOR1 Single N-Glycosylation Site Mutants Are Functionally Expressed in Insect Cells

**FIGURE 3.**
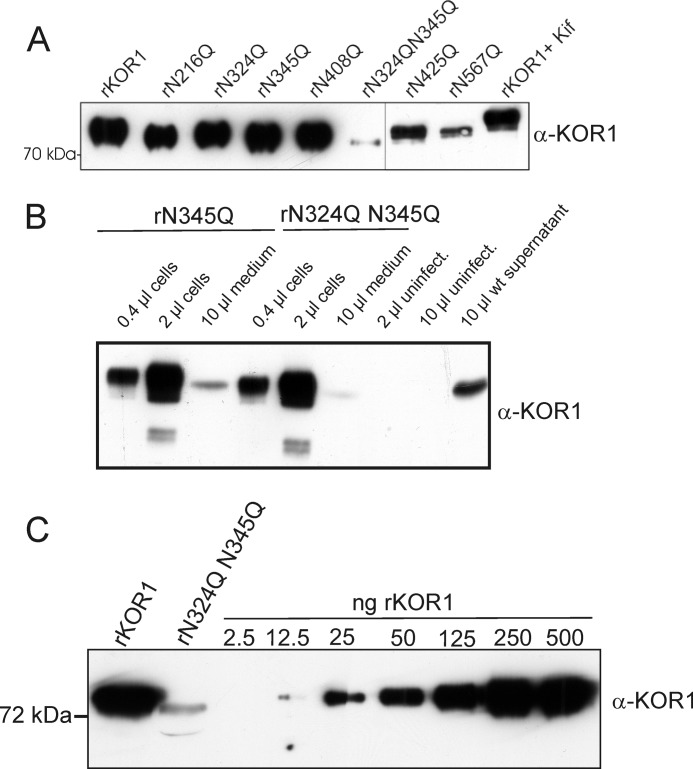
**Comparison of rKOR1 WT and *N*-glycosylation mutant expression in insect cells.** *A*, the indicated recombinant KOR1 variants were expressed in insect cells using a baculovirus expression system and purified from cell supernatants by means of affinity chromatography. Equal volumes were separated by SDS-PAGE and analyzed by immunoblotting. *B*, comparison of expression levels in cell extracts (cells) and cell supernatants (medium) of rKOR1 N345Q and the double mutant rKOR1 N324Q/N345Q. Extracts of uninfected cells were included as negative control, and supernatant expressing rKOR1 WT was included as positive control. *C*, comparison of rKOR1 WT and rKOR1 N324Q/N345Q expression levels in cell supernatants. 10 μl of cell supernatant from cells expressing rKOR1 WT and rKOR1 N345Q/N345Q were separated by SDS-PAGE and analyzed by immunoblotting with anti-KOR1 antibodies. *Kif*, kifunensine.

To examine the impact of individual *N*-glycans on rKOR1 expression, stability, and activity, selected glycosylation sites were eliminated by site-directed mutagenesis where asparagine was changed to glutamine. *N*-Glycosylation sites Asn-216, Asn-324, and Asn-345 were chosen due to their proximity to the proposed catalytic cleft of KOR1 (18). In addition, glycosylation sites Asn-408, Asn-425, and Asn-567 were mutated because they are located in the catalytic domain of KOR1 and mostly also present in KOR1 orthologs from other species. The first two sites (Asn-108 and Asn-133) that are close to the transmembrane domain of native *A. thaliana* KOR1 were not mutated because they are not evolutionarily conserved (18–20). To further investigate the role of glycosylation for KOR1 expression and stability, we generated a double mutant (N324Q/N345Q). All of the single *N*-glycosylation mutants were efficiently expressed and could be purified from the culture medium. However, the level of secretion was lower for rKOR1 N567Q than for the other single mutants (Fig. 3*A*), although the rKOR1 content of the respective cell extracts was similar (data not shown). This suggests that absence of the *N*-glycan attached to Asn-567 interferes with proper folding of the enzyme in the endoplasmic reticulum of the insect cells in a subtle manner. In contrast, the amount of secreted rKOR1 N324Q/N345Q was strongly reduced as compared with wild-type and rKOR1 N345Q (Fig. 3, *B* and *C*), indicating that folding of the double mutant was heavily impaired. LC-ESI-MS analysis of purified rKOR1 forms confirmed that all single-mutant proteins lacked only the *N*-glycan usually attached to the mutated asparagine residue (data not shown).

##### Removal of Individual N-Glycosylation Sites Affects KOR1 Activity

We chose to assay enzymatic activity of rKOR1 WT and the mutant variants using carboxymethylcellulose ((carboxymethyl)cellulose 4M) as substrate and a reducing sugar assay to monitor the released glucose. In previous studies, (carboxymethyl)cellulose 4M was proven to be the best substrate, when KOR1 orthologs were analyzed with different known cellulose derivatives (18, 20). rKOR1 WT and the mutated variants hydrolyzed (carboxymethyl)cellulose 4M, albeit to a varying extent. rKOR1 N408Q and rKOR1 N425Q displayed similar hydrolysis rates as rKOR1 WT, indicating that these two *N*-glycosylation sites are not critical for maximal enzymatic activity. Mutations at glycosylation sites Asn-216, Asn-324, and Asn-345 reduced KOR1 enzymatic activity by 15–30%. Removal of the oligomannosidic *N*-glycan on glycosylation site Asn-567 resulted in the most pronounced difference with ∼50% of wild-type activity (Table 1).

**TABLE 1:** **Enzymatic activity of rKOR1 WT and mutated rKOR1 forms using (carboxymethyl)cellulose 4M as substrate** Data are expressed as means ± S.D. of 2–7 experiments. KIF, kifunensine.

rKOR1 protein	Enzyme activity
	*nmol min*^−*1*^ *mg*^−*1*^
rKOR1 WT	374 ± 18
rN216Q	244 ± 25*^a^*
rN324Q	307 ± 8*^a^*
rN345Q	271 ± 25*^a^*
rN408Q	379 ± 18
rN425Q	394 ± 34
rN567Q	200 ± 15*^a^*
rKOR1 KIF	402 ± 40

*^a^ p* < 0.05 as compared to rKOR1 WT.

To ensure that the effect on KOR1 activity is not a matter of protein instability due to the loss of individual *N*-glycans, rKOR1 WT and mutated forms were incubated at 30 °C over a time range from 30 to 240 min prior to activity assays. rKOR1 WT and all single mutants except for N425Q retained at least 90% of their enzymatic activity after preincubation for 120 min (data not shown). In contrast, the activity of rKOR1 N425Q was found to be reduced to 69% (120 min) or 44% (240 min). The sensitivity of the activity assay was tested using varying amounts of purified proteins. At least 350 ng of recombinant protein per assay were needed to obtain reliable results. The hydrolysis rate of (carboxymethyl)cellulose 4M did increase in a linear manner up to 4 μg of enzyme protein used per assay.

##### Enzymatic Deglycosylation Results in Reduced KOR1 Activity

**FIGURE 4.**
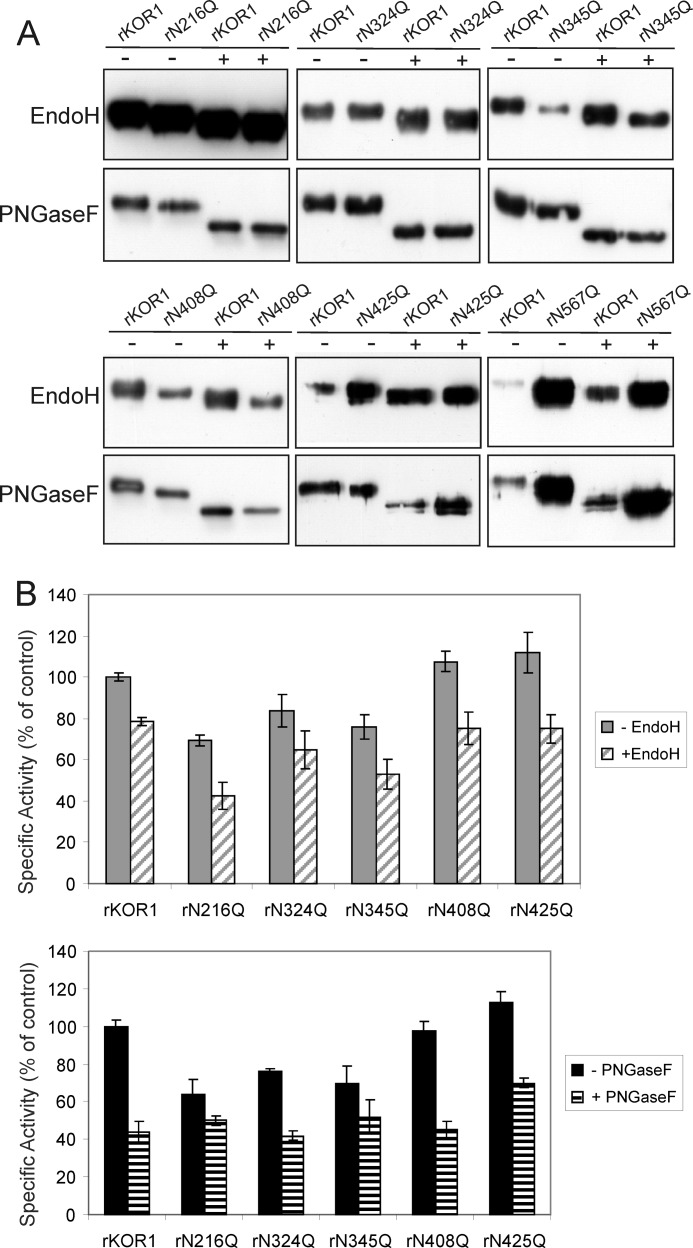
**rKOR1 WT and single *N*-glycosylation mutants display reduced enzymatic activity upon deglycosylation with Endo H and PNGase F.** *A*, rKOR1 variants were expressed in insect cells, purified, and subjected to enzymatic deglycosylation with Endo H and PNGase F under non-denaturing conditions followed by immunoblotting with anti-KOR1 antibodies. *B*, digested and non-digested samples were subjected to activity assays with (carboxymethyl)cellulose 4M as substrate. Data are expressed as mean ± S.D. of three independent experiments and presented as percentage of the specific activity of untreated rKOR1 WT (control). It was not possible to perform these assays with the rKOR1 N567Q mutant due to insufficient amounts of purified enzyme.

Previously, it was shown that enzymatic deglycosylation of the heterologously produced *B. napus* and *P. tremula x tremuloides* KOR1 orthologs caused an almost complete loss of enzymatic activity (18, 20). To test the impact of deglycosylation on the activity of the *A. thaliana* enzyme, rKOR1 WT and mutant forms were treated with PNGase F and Endo H under non-denaturing conditions. The extent of deglycosylation was monitored by immunoblotting with anti-KOR1 antibodies. For all analyzed rKOR1 mutants, a reduction in molecular mass was observed (Fig. 4*A*). In accordance with data for rKOR1 WT, the shift in mobility was more pronounced after treatment with PNGase F. The slight shift of rKOR1 N567Q in the Endo H-digested sample results very likely from removal of minor amounts of oligomannosidic *N*-glycans present on the other seven *N*-glycosylation sites.

Upon deglycosylation with PNGase F, rKOR1 displayed ∼50% residual enzymatic activity (Fig. 4*B*), whereas Endo H treatment of rKOR1 resulted in a loss of 20–30% compared with the initial enzymatic activity (Fig. 4*B*). Interestingly, the specific activity of the single mutants N216Q, N324Q, N345Q, N408Q, and N425Q after deglycosylation with PNGase F was at least as high as that of deglycosylated rKOR1 WT. The activities of glycosylation mutants treated with Endo H were comparable with that of Endo H-digested rKOR1 WT, being reduced by 20–30% as compared with the undigested forms.

**FIGURE 5.**
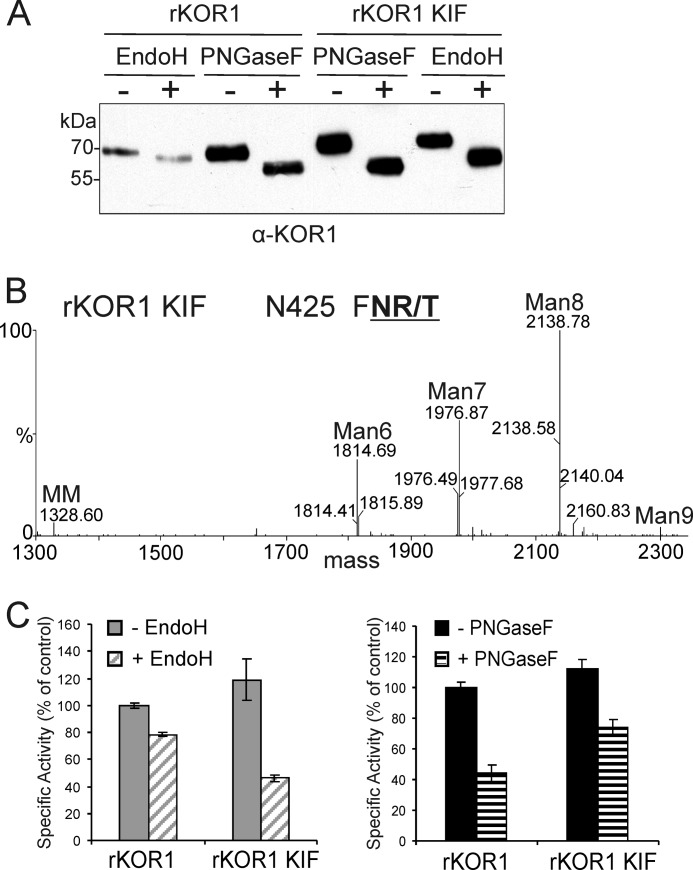
**Recombinant KOR1 with predominantly oligomannosidic *N*-glycans is stably expressed in insect cells and fully active.** rKOR1 WT was expressed in insect cells in the absence or presence of kifunensine (*KIF*), purified, and subjected to PNGase F or Endo H treatment. *A*, immunoblot analysis with anti-KOR1 antibodies after enzymatic deglycosylation under denaturing conditions. *B*, LC-ESI-MS analysis of rKOR1 WT expressed in the presence of kifunensine (rKOR1 *KIF*). The glycosylation pattern of the peptide containing Asn-425 is depicted. The *N*-glycosylation site is shown in *bold* and *underlined. MM*, Man_3_GlcNAc_2_; *Man6*, Man_6_GlcNAc_2_; *Man7*, Man_7_GlcNAc_2_; *Man8*, Man_8_GlcNAc_2_; *Man9*, Man_9_GlcNAc_2_. *C*, activity assays were performed using (carboxymethyl)cellulose 4M as substrate after enzymatic deglycosylation under non-denaturing conditions. Data are expressed as mean ± S.D. of three independent experiments and presented as percentage of the specific activity of untreated rKOR1 WT (control).

To analyze the significance of the observed *N*-glycosylation profile for rKOR1 activity, we expressed rKOR1 WT in insect cells in the presence of kifunensine (rKOR1 KIF), a class I α-mannosidase inhibitor that prevents processing of oligomannosidic glycans in the endoplasmic reticulum. As a consequence of kifunensine treatment, all eight *N*-glycans on rKOR1 should contain Man_5–9_GlcNAc_2_ oligosaccharides instead of the paucimannosidic structures detected on the wild-type enzyme. Immunoblot analysis and subsequent affinity purification showed that rKOR1 KIF was present in the culture supernatant in amounts comparable with untreated rKOR1 WT (Fig. 3*A*). The reduced mobility on SDS-PAGE indicated that rKOR1 KIF indeed contains largely unprocessed oligomannosidic *N*-glycans (Fig. 5*A*). LC-ESI-MS analysis of glycopeptides from rKOR1 KIF confirmed the presence of predominantly oligomannosidic *N*-glycans (Man_7_GlcNAc_2_ to Man_9_GlcNAc_2_) (Fig. 5*B* and data not shown). Notably, the enzymatic activity of rKOR1 KIF was indistinguishable from rKOR1 WT (Table 1), indicating that processing of *N*-glycans from oligomannosidic to paucimannosidic structures is not required for KOR1 activity. Interestingly, the activity of rKOR1 KIF was more sensitive to Endo H than that of the wild-type enzyme. As expected, this was not observed upon treatment with PNGase F (Fig. 5*C*). Collectively, these data suggest that *N*-glycosylation is important for KOR1 activity, but a distinct *N*-glycan structure is not required for its function.

##### KOR1 Expressed in N. benthamiana Carries Complex and Oligomannosidic N-Glycans

**FIGURE 6.**
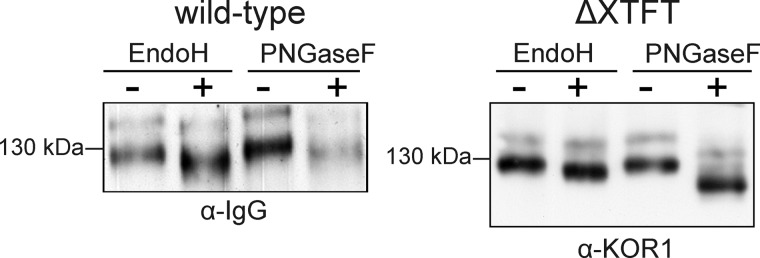
**KOR1-Fc-GFP is *N*-glycosylated.** KOR1-Fc-GFP was transiently expressed in *N. benthamiana* wild-type or ΔXTFT plants, purified, and digested with Endo H or PNGase F and then subjected to immunoblot analysis with anti-human IgG or anti-KOR1 antibodies. Deglycosylation experiments with purified KOR1-Fc-GFP were performed under denaturing conditions.

**FIGURE 7.**
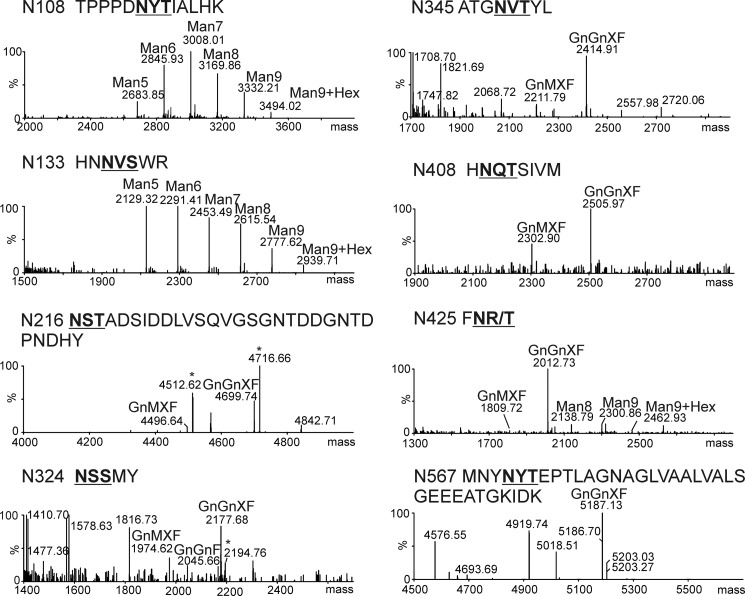
**KOR1-Fc-GFP is glycosylated with oligomannosidic and complex *N*-glycans when transiently expressed in *N. benthamiana* wild-type plants.** LC-ESI-MS analyses of all eight KOR1 glycopeptides. *N*-glycosylation sites are shown in *bold* and *underlined*. The *asterisks* denote the presence of ammonium adducts. *GnGnXF*, GlcNAc_2_XylFucMan_3_GlcNAc_2_; *GnMXF*, GlcNAcXylFucMan_3_GlcNAc_2_; *Man5*, Man_5_GlcNAc_2_; *Man6*, Man_6_GlcNAc_2_; *Man7*, Man_7_GlcNAc_2_; *Man8*, Man_8_GlcNAc_2_; *Man9*, Man_9_GlcNAc_2_; *N108*, Asn-108; *N133*, Asn-133; *N216,* Asn-216; *N324,* Asn-324; *N345,* Asn-345; *N408,* Asn-408; *N425,* Asn-425; *N567,* Asn-567.

Next, we attempted to analyze the glycosylation pattern of KOR1 expressed in plants. To this end, we transiently expressed the full-length KOR1 protein, including its cytoplasmic region and single transmembrane domain in *N. benthamiana* leaf epidermal cells. To facilitate purification and subcellular localization, the Fc domain of the human IgG1 heavy chain and GFP were fused to the C-terminal end of KOR1. To distinguish between complex and oligomannosidic *N*-glycans, KOR1-Fc-GFP was expressed in *N. benthamiana* wild-type and ΔXTFT lines (31) prior to treatment with Endo H and PNGase F and analysis by immunoblotting with anti-KOR1 antibodies. The complex *N*-glycans produced by the ΔXTFT line are almost completely devoid of core α1,3-fucose residues and thus sensitive to digestion by PNGase F. KOR1-Fc-GFP produced in these plants was sensitive to Endo H as well as PNGase F. However, the digested polypeptides displayed different migration behavior indicative of the simultaneous presence of oligomannosidic and complex *N*-glycans (Fig. 6). On immunoblots, intense 130-kDa bands as well as faint bands with a molecular mass of ∼160 kDa were detected. Immunoblot analysis with anti-GFP antibody revealed that the upper band represents the full-length KOR1-Fc-GFP protein (data not shown), whereas the lower band corresponds to KOR1-Fc lacking the fluorescent protein tag. The glycan structures of KOR1 from the more abundant lower band were analyzed by mass spectrometry. In accordance with data from the insect cell-derived rKOR1, all eight *N*-glycosylation sites of the extracellular domain were occupied with glycans. *N*-Glycosylation sites Asn-108 and Asn-133 carried mainly oligomannosidic N-glycans, whereas all other sites showed processed complex *N*-glycans with GlcNAc_2_Man_3_GlcNAc_2_ (GnGnXF) as predominant glycoform (Fig. 7).

**FIGURE 8.**
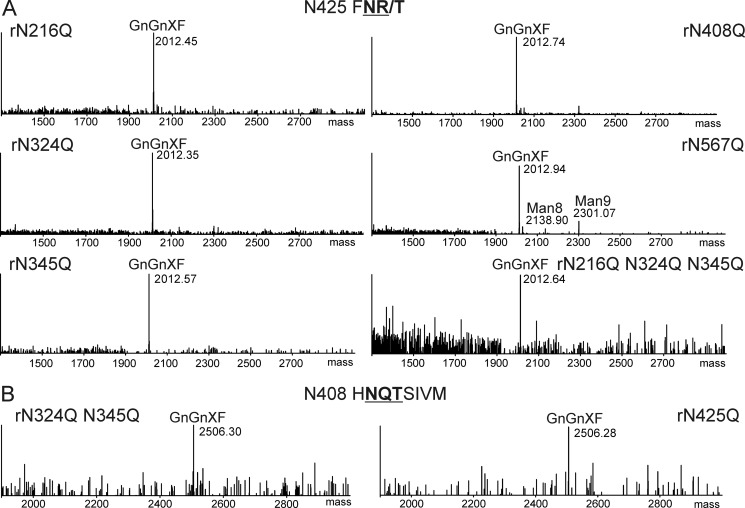
**KOR1 *N*-glycosylation mutants contain complex *N*-glycans when expressed in plants.** *A*, LC-ESI MS analyses of the glycopeptide containing glycosylation site Asn-425. *B*, LC-ESI MS analyses of the glycopeptide containing glycosylation site Asn-408. All KOR1-Fc-GFP *N*-glycosylation mutant variants were transiently expressed in *N. benthamiana* wild-type plants, purified, and subjected to MS analysis. *N*-glycosylation sites are shown in *bold* and *underlined. GnGnXF*, GlcNAc_2_XylFucMan_3_GlcNAc_2_; *GnMXF*, GlcNAcXylFucMan_3_GlcNAc_2_; *Man5*, Man_5_GlcNAc_2_; *Man6*, Man_6_GlcNAc_2_; *Man7*, Man_7_GlcNAc_2_; *Man8*, Man_8_GlcNAc_2_; *Man9*, Man_9_GlcNAc_2_; *N408*, Asn-408; *N425,* Asn-425.

To determine whether individual *N*-glycans contribute to expression and subcellular targeting of KOR1 in plants, we introduced all *N*-glycosylation site mutations described above also into KOR1-Fc-GFP. Furthermore, a triple mutant (N216Q/N324Q/N345Q) was generated, which lacks all *N*-glycosylation sites in the potential catalytic cleft of KOR1. All detectable glycopeptides derived from the mutant variants displayed a similar glycosylation profile as observed for wild-type KOR1 (Fig. 8, *A* and *B*). These findings demonstrate that the *N*-glycans of the wild-type enzyme and the single, double, and triple mutants tested are all properly processed in the plant Golgi. However, enzymatic analysis of wild-type and mutant KOR1-Fc-GFP could not be performed due to insufficient amounts of purified protein.

##### Endogenous A. thaliana KOR1 Is Glycosylated with Oligomannosidic and Complex N-Glycans

**FIGURE 9.**
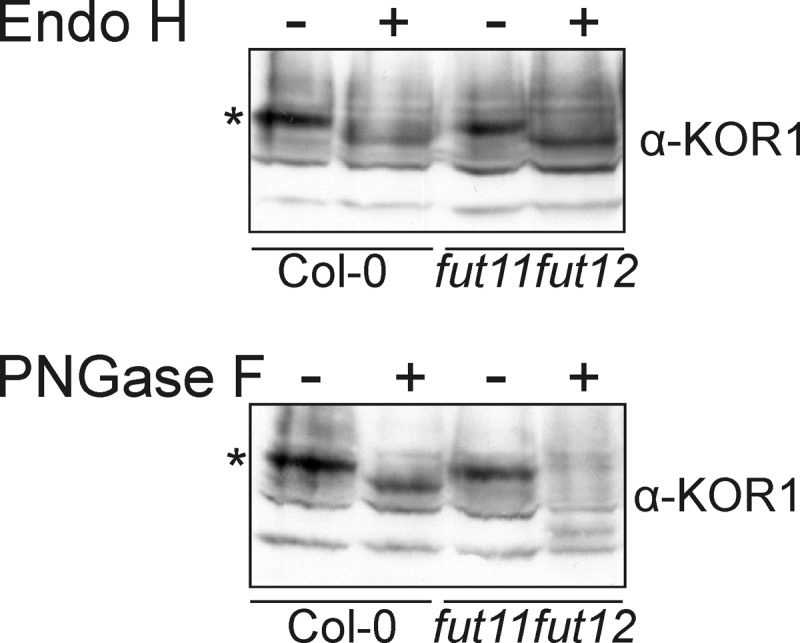
**Endogenous *A. thaliana* KOR1 protein is sensitive to endoglycosidase treatment.** Protein extracts of wild-type and *fut11 fut12* seedlings were digested with Endo H and PNGase F, subjected to SDS-PAGE, and analyzed by immunoblotting with anti-KOR1 antibodies. The position of the undigested KOR1 band is marked by an *asterisk*.

Expression of KOR1-Fc-GFP in *N. benthamiana* showed the presence of oligomannosidic and complex *N*-glycans. To investigate the glycosylation of endogenous *A. thaliana* KOR1, proteins extracted from *A. thaliana* Col-0 seedlings as well as from the *fut11 fut12* mutant (27), which generates complex *N*-glycans lacking core α1,3-fucose were incubated in the presence or absence of Endo H and PNGase F and then subjected to immunoblotting with anti-KOR1 antibodies. In both genetic backgrounds, small shifts in mobility were observed after Endo H treatment indicating the presence of oligomannosidic *N*-glycans. In the *fut11 fut12* line, PNGase F digestion resulted in a mobility shift that was more pronounced than in wild-type plants (Fig. 9), indicating that most of the *N*-glycosylation sites of endogenous KOR1 are decorated with complex *N*-glycans carrying core α1,3-fucose residues. In summary, these data are fully in accordance with the *N*-glycan structures detected on KOR1-Fc-GFP transiently expressed in *N. benthamiana*.

##### KOR1 G429R Contains only Oligomannosidic N-Glycans

**FIGURE 10.**
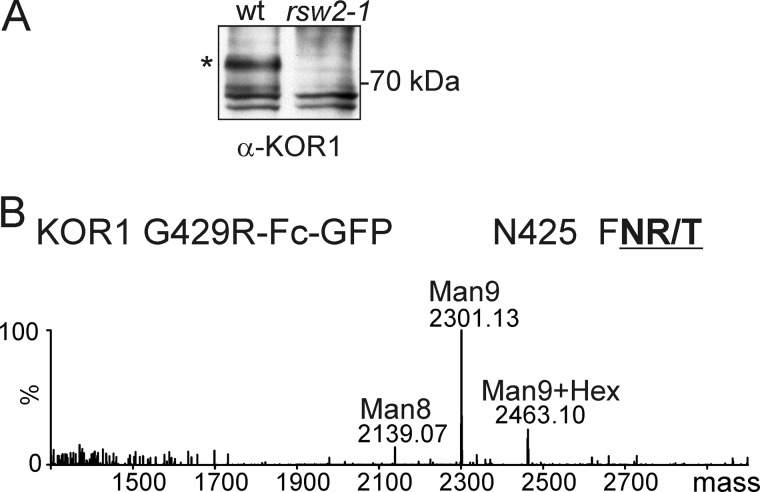
**KOR1 G429R-Fc-GFP expressed in *N. benthamiana* carries oligomannosidic *N*-glycans.** *A*, immunoblot analysis of protein extracts from Col-0 wild type (wt) and *rsw2–1* seedlings. The position of the wild-type KOR1 band is marked by an *asterisk. B*, KOR1 G429R-Fc-GFP was purified from *N. benthamiana* leaves and subjected to LC-ESI-MS analysis. Glycosylation of the peptide containing Asn-425 is depicted. The *N*-glycosylation site is shown in *bold* and *underlined. Man8*, Man_8_GlcNAc_2_; *Man9*, Man_9_GlcNAc_2_; *N425*, Asn-425.

The phenotype of the *rsw2-1* mutant is caused by a point mutation in the KOR1 gene leading to an amino acid change from glycine to arginine (KOR1 G429R) (14). The described additive growth phenotype of the *cgl1 rsw2-1* double mutant suggests that the *N*-glycans of *rsw2-1* KOR1 are usually processed to complex *N*-glycans in the Golgi (21). The amounts of endogenous KOR1 in *rsw2-1* seedlings are quite low, precluding direct analysis of its *N*-glycosylation status (Fig. 10*A*). Consequently, to investigate the nature of the *N*-glycans on KOR1 G429R, we expressed a KOR1 G429R-Fc-GFP variant in *N. benthamiana*, purified the protein, and analyzed its glycopeptides by LC-ESI-MS. Although the obtained amounts of purified KOR1 G429R-Fc-GFP were considerably lower than for KOR1-Fc-GFP, we could identify four of the eight glycopeptides. The identified glycopeptides displayed a predominant peak corresponding to the mass of Man_9_GlcNAc_2_, with additional peaks corresponding to Man_8_GlcNAc_2_ and Glc_1_Man_9_GlcNAc_2_ (Fig. 10*B* and data not shown). The presence of oligomannosidic structures and glucose-containing oligosaccharides instead of complex *N*-glycans indicates that KOR1 G429R-Fc-GFP is retained by quality control processes in the ER and hence does not reach the Golgi. Collectively, these data strongly suggest that the severe phenotype of *cgl1 rsw2-1* is not caused by an *N*-glycan processing defect that directly affects KOR1 glycosylation.

## DISCUSSION

*N*-Glycosylation is a major co- and post-translational modification in plants and the majority of all proteins destined for different compartments of the endomembrane pathway are glycosylated. The *N*-glycan biosynthesis and processing pathways of *A. thaliana* are already quite well understood (36, 37), and it has been shown that *N*-glycosylation is crucial for plant development (3, 23, 38, 39) and plays an important role in tolerance and response to biotic and abiotic stress (21, 22, 40–46). However, the analysis of the *N*-glycoproteome and the *N*-glycosylation state of individual glycoproteins from *A. thaliana* and other plant species is still in its infancy (34, 47–49). In this study, we investigated the *N*-glycans of *A. thaliana* KOR1 and their role for protein stability and enzymatic activity.

Overall, our data for recombinant insect cell-derived *A. thaliana* KOR1 are consistent with enzyme activity assays reported in previous studies for KOR1 orthologs (18, 20) and confirm that *N*-glycosylation of KOR1 is important for its activity. In contrast to the previous findings, where 80% and more of the enzymatic activity was lost upon deglycosylation we could still detect a considerable amount of residual enzymatic activity in our study. In addition, our data suggest that different *N*-glycans contribute to optimal KOR1 enzymatic activity and proper folding of KOR1 when recombinantly expressed in insect cells. Individual mutation of Asn-216, Asn-324, Asn-345, and Asn-567 into glutamine resulted in considerably lower enzymatic activity. In the case of Asn-216, Asn-324, and Asn-345, this effect could be due to the proximity of these residues to the putative active site of KOR1. However, this does not apply to Asn-567. Because removal of the latter *N*-glycosylation site also resulted in reduced secretion of the recombinant enzyme, it appears likely that absence of the *N*-glycan attached to Asn-567 interferes with proper folding of KOR1 rather than exerting a direct effect on its enzymatic activity.

Notably, the effects of the different *N*-glycosylation site mutations on KOR1-Fc expression in plants is less pronounced. For example, the double mutant lacking *N*-glycans on two conserved KOR1 *N*-glycosylation sites (Asn-324, Asn-345) could not be efficiently expressed in insect cells, whereas the full-length KOR1-Fc-GFP variant with the same mutations was produced in comparable amounts as wild-type KOR1 in *N. benthamiana*. The presence of complex *N*-glycans demonstrates further that this mutant variant is processed in the plant Golgi, suggesting normal transport through the endomembrane system. In future studies, it remains to be shown whether the different *N*-glycan mutants can complement *kor1* mutants and whether *N*-glycosylation of KOR1 is required for its function in plants.

We attempted to purify native KOR1 from *A. thaliana* seedlings using affinity purification with different antibodies against KOR1. However, despite numerous efforts, it was not possible to enrich sufficient amounts of endogenous KOR1 to perform glycosylation site analysis by mass spectrometry. Consequently, we transiently expressed a KOR1 fusion protein in leaves of *N. benthamiana* for analysis of its *N*-glycan composition when produced *in planta*. In accordance with data from endoglycosidase treatment and immunoblotting of endogenous KOR1 from *A. thaliana* seedlings, we detected oligomannosidic as well as processed complex *N*-glycans. Notably, we observed minor differences between *N*-glycans present on recombinant KOR1 produced in insect cells and KOR1 expressed in *N. benthamiana* leaf epidermal cells. These differences are found on the first two and the last *N*-glycosylation sites and are very likely caused by the presence/absence of different protein domains. In insect cells, we expressed a soluble KOR1 variant lacking the N-terminal cytoplasmic region and the single transmembrane domain to obtain a secreted form of the protein that can be readily purified from cell supernatants and used for *in vitro* activity assays. These constructs were designed to allow a direct comparison with previous studies relying on recombinant enzymes produced in *P. pastoris* (18, 20). In plants, we expressed full-length KOR1 fused to GFP and the Fc domain of human IgG. Although we cannot rule out that the protein tags for purification and localization influence protein folding and/or accessibility of individual glycosylation sites, it is more likely that the major effect comes from the membrane anchoring of KOR1. The first two *N*-glycan sites of KOR1 are very close to the transmembrane domain and consequently might not be as accessible for processing as sites that are more exposed in the catalytic domain. This proximity to the membrane could explain the presence of partially or unprocessed oligomannosidic *N*-glycans on Asn-108 and Asn-133. In contrast, these two sites are probably accessible (and thus modified with paucimannosidic *N*-glycans) in the variant produced in insect cells, which lacks the 90 most N-terminal amino acid residues.

Furthermore, we cannot exclude that KOR1 folding and ER-mediated quality control are different in insect cells and in plants. The presence/absence of certain molecular chaperones, lectins, or folding catalysts could contribute to the observed differences in *N*-glycan maturation as well as the different fate of the double *N*-glycosylation site mutant. Plants contain, for example, two calnexin and three calreticulin proteins, and calreticulin 3 seems specific for plants and has been found to play an important role in ER quality control processes of heavily glycosylated leucine-rich repeat receptor kinases (43, 44, 50).

The finding that the *A. thaliana* mutants *cgl1* and *mns3*, which both harbor alterations in complex *N*-glycan formation, enhance the growth phenotype of the temperature-sensitive *rsw2-1* allele even at the permissive temperature (21, 23) led to the hypothesis that processing of KOR1 *N*-glycans in the Golgi could play a direct role for KOR1 function (21, 24). Block of *N*-glycan maturation in the *cgl1* mutant causes the accumulation of Man_5_GlcNAc_2_ structures because all subsequent *N*-glycan processing reactions leading to the formation of hybrid and complex *N*-glycans depend on GnTI activity, which is impaired in this mutant (51–53). The *mns3* mutant accumulates mainly Man_6_GlcNAc_2_ and displays considerably reduced amounts of GnGnXF, the most common complex *N*-glycan in *A. thaliana* leaves and seedlings (23). Our results for the recombinant KOR1 variant carrying only oligomannosidic *N*-glycans indicates that *N*-glycan processing in the Golgi is not important for KOR1 enzymatic activity. As a consequence, it is possible that the complex *N*-glycan processing defects in *cgl1* and *mns3* do not directly affect the KOR1 protein variant present in *rsw2-1* but have additional as yet unidentified glycoprotein targets that could be important for KOR1 function(s) *in planta.* The presence of oligomannosidic *N*-glycans on KOR1-G429R-Fc-GFP (which is indicative of ER retention) further corroborates this finding. The observed additive phenotype of *cgl1 rsw2-1* and *mns3 rsw2-1* could be caused by a combination of the KOR1 defect in *rsw2-1* and the *N*-glycan processing defect involving one or several other glycoproteins that could (but not necessarily have to) act in the same cellular pathway (cellulose synthesis). Alternatively, the less severe phenotype of *rsw2-1* compared with other *kor1* mutants (14, 17) indicates that a small amount of KOR1 G429R escapes from the ER quality control process and is targeted through the Golgi to its final destination, where it is partially or fully functional. In this scenario, altered *N*-glycan processing in *mns3* and *cgl1* could still directly affect the subcellular localization or function of KOR1 G429R.

Further studies such as the combination of *rsw2-1* with other *N*-glycosylation and *N*-glycan processing mutants (27, 54, 55) and the characterization of additional glycoproteins are necessary to finally understand the role of complex *N*-glycans in cellulose biosynthesis.

## Footnotes

2 The abbreviations used are:

ER: endoplasmic reticulum
LC-ESI-MS: liquid chromatography-electrospray ionization mass spectrometry
ΔXTFT: *N. benthamiana* glycosylation mutant deficient in β1,2-xylosyltransferase and core α1,3-fucosyltransferase
KOR1: *A. thaliana* KORRIGAN1
rKOR1: recombinant KOR1
Endo H: endoglycosidase H
PNGase F: peptide *N*-glycosidase F
KIF: kifunensine.
